# Supplementary material for: Wheat Pm55 alleles exhibit distinct interactions with an inhibitor to cause different powdery mildew resistance
Source: Nat Commun. 2024 Jan 13;15:503. doi: 10.1038/s41467-024-44796-0 (PMC10787760; doi:10.1038/s41467-024-44796-0)
Supplement: Supplementary file 1 — Supplementary Information [file 41467_2024_44796_MOESM1_ESM.pdf]

Wheat *Pm55* alleles exhibit distinct interactions with an inhibitor to cause different powdery mildew resistance

Lu *et al.*

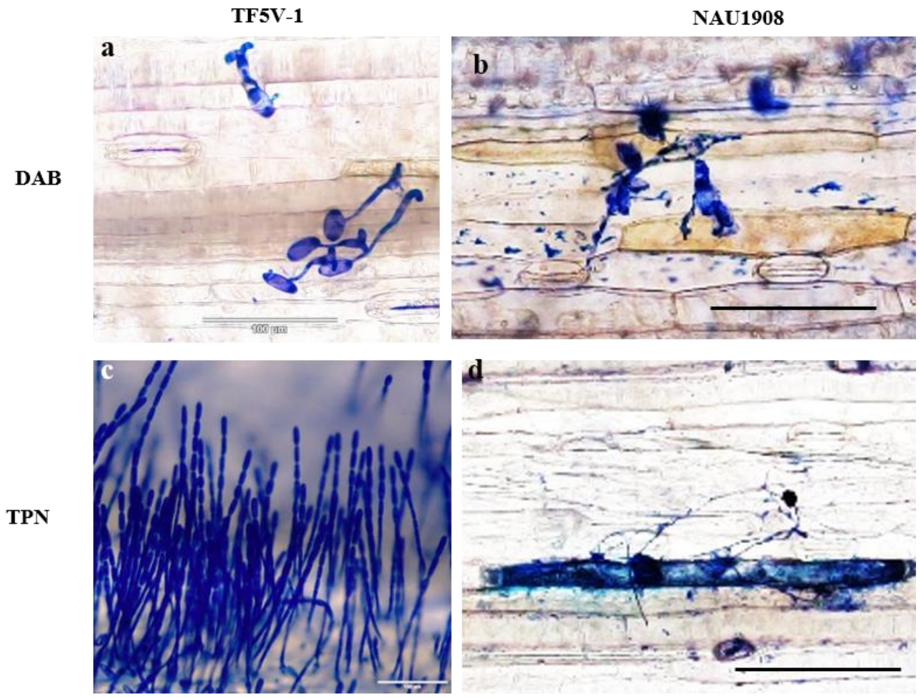

Supplementary Fig. 1. Microscopy of *Bgt* hyphal development on leaves of TF5V-1 and NAU1908 at seedling stage planted in greenhouse under high inoculum density.

a,b DAB staining of TF5V-1 (**a**) and NAU1908 (**b**) leaves infected with *Bgt* isolate E09 at 2 dpi. Brown staining shows the accumulation of H_2O_2. **c,d** Trypan blue staining of TF5V-1 (**c**) and NAU1908 (**d**) leaves infected with *Bgt* isolate E09 at 7 dpi to visualize fungal structures and plant cell death. These results exhibited that TF5V-1 carrying *Pm55* is highly susceptible to *Bgt* E09 at seedling stage, but NAU1908 carrying *Pm5V* is highly resistant. Scale bars, 100 μm.

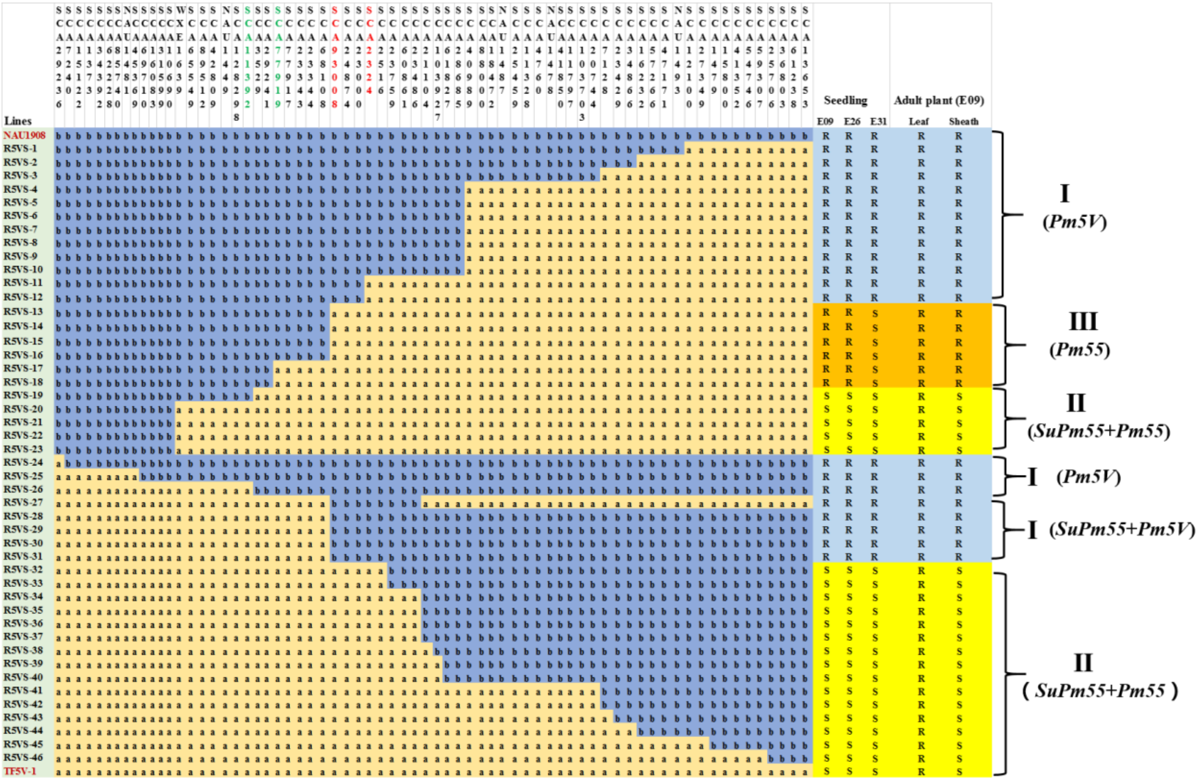

Supplementary Fig. 2. The genotypes and phenotypes of a panel of 46 recombinant lines derived from TF5V-1 × NAU1908.

Type I recombinant lines show the parental NAU1908 phenotypes. Type II recombinant lines show the parental TF5V-1 phenotypes. Type III recombinant lines exhibit seedling resistance to *Bgt* isolates E09 and E26 but susceptible to E31, which are distinct phenotypes from parents NAU1908 and TF5V-1. All lines show resistance to E09 at adult-plant stage, but Type II lines have the leaf sheath susceptibility. Detailed molecular markers information is provided in Supplementary Data 1.

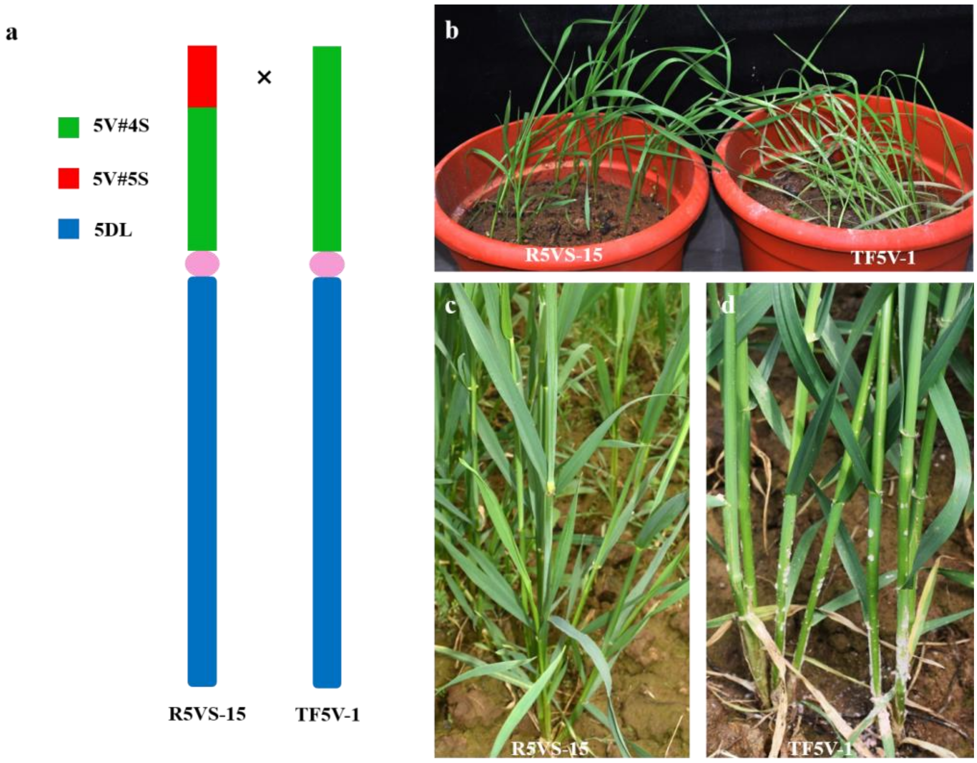

Supplementary Fig. 3. Crossing between R5VS-15 and TF5V-1 with distinct powdery mildew responses.

a R5VS-15 is a type III recombinant T5DL·5VS line carrying chromosome segments of 5V#4S and 5V#5S simultaneously. **b** Seedling responses of parents R5VS-15 (R) and TF5V-1(S) to *Bgt* isolate E09 at seedling stage. **c,d** Adult responses of parents R5VS-1 (**c**) and TF5V-1 (**d**) to *Bgt* isolate E09. R5VS-15 showed resistance on all tissues and all stages, whereas TF5V-1 exhibited adult-plant resistance with lower leaf sheath susceptibility.

Lines	NAU1449	SCA11392	SCA4218	SCA3806	SCA15749	SCA39816	SCA3529	SCA29241	Xdv14	SCA77919	Seedling	Adult plant	
											E09	Leaf	Sheath
R5VS-15	b	b	b	b	b	b	b	b	b	b	R	R	R
F_3-94-2	b	b	b	a	a	a	a	a	a	a	S	R	S
F_3-167-5	b	b	b	b	b	a	a	a	a	a	R	R	R
F_3-2008-13	b	b	b	b	b	b	b	a	a	a	R	R	R
F_3-1194-7	b	b	b	b	b	b	b	b	a	a	R	R	R
F_3-903-11	b	b	b	b	b	b	b	b	b	a	R	R	R
F_3-2074-6	b	b	b	b	b	b	b	b	b	a	R	R	R
TF5V-1	a	a	a	a	a	a	a	a	a	a	S	R	S

Supplementary Fig. 4. Fine mapping of *SuPm55* using six new recombinant lines.

Powdery mildew responses of recombinant plants to *Bgt* isolate E09 at both seedling and adult-plant stages are shown at the right. The green color indicates the segments of chromosome arm 5V#4S. Red color indicates the segments of chromosome arm 5V#5S. Inhibitor locus was further narrowed to the interval flanked by InDel markers *SCA4218* and *SCA39816*. The detailed information of the five markers, *SCA428*, *SCA3806*, *SCA15749*, *SCA39816* and *Xdv-14* developed in this study is provided in Supplementary Data 2.

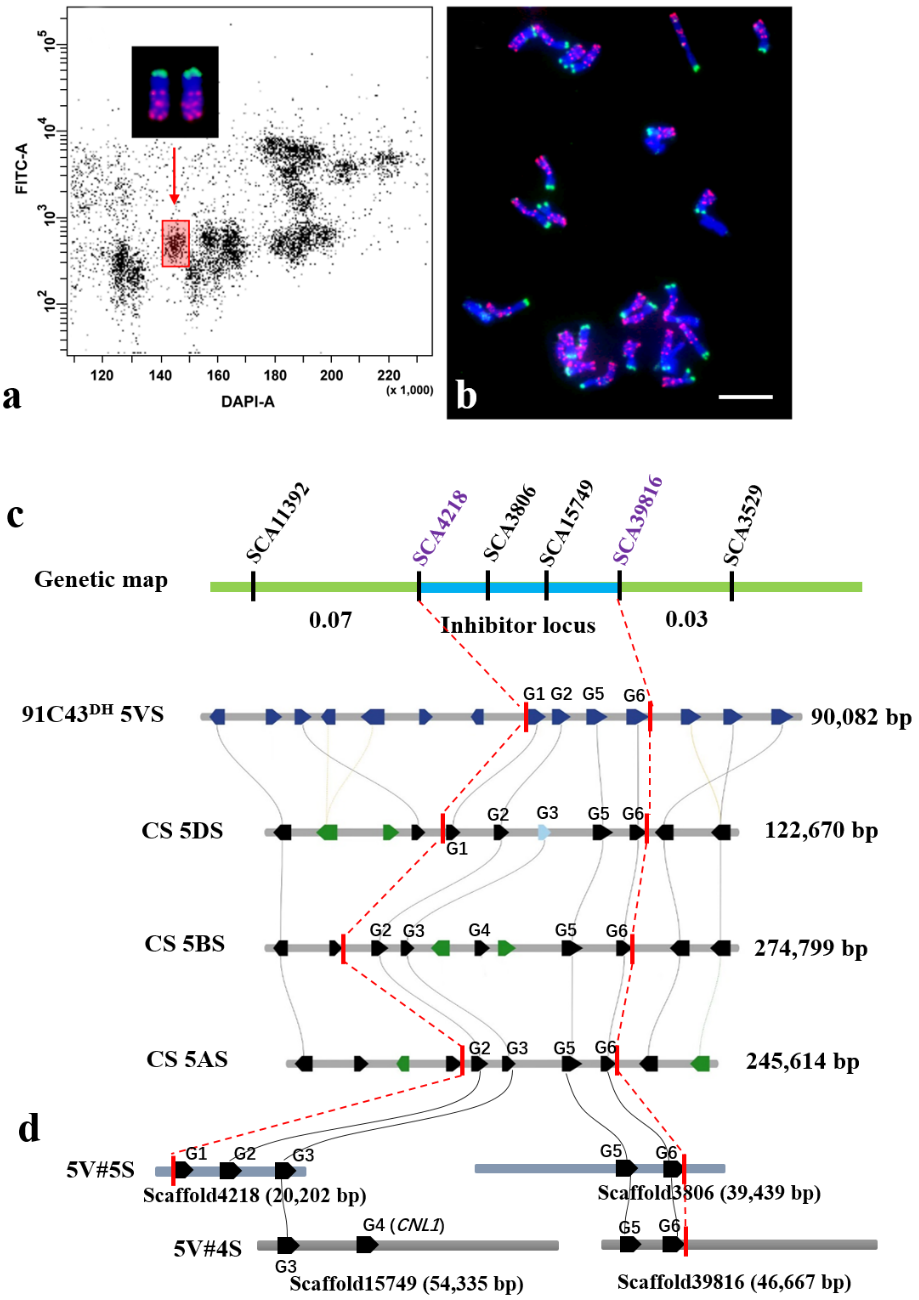

Supplementary Fig. 5. Flow-sorting translocated chromosome T5DL·5V#4S and isolating candidate genes in the inhibitor region on chromosome arm 5V#4S.

a Flow cytometric sorting chromosome T5DL·5V#4S from TF5V-1. Bivariate flow karyotype FITC fluorescence pulse area (FITC-A) vs. DAPI fluorescence pulse area (DAPI-A) of the chromosomes labeled by FISHIS with FITC-conjugated probe for GAA microsatellites and stained by DAPI. Translocated chromosome T5DL·5V#4S was sorted using the sort window shown as red rectangle at average purity of 80.8%. Inset: images of the flow sorted translocated chromosome T5DL·5V#4S. **b**

Example of T5DL·5V#4S translocated chromosomes sorted onto a microscope slide after FISH with probes for pSc119.2 repeat (green), Afa family repeat (red) and 45S rDNA (yellow); chromosomes were counterstained with DAPI (blue). Bar =10 µm. **c** The collinearity analysis of the inhibitor physical region on chromosome arm 5VS of 91C43^{DH} with Chinese Spring 5AS, 5BS and 5DS. This physical region on 5VS of 91C43^{DH} is 90,082 bp, including four annotated genes, whereas the inhibitor regions on CS 5DS, 5BS and 5AS respectively include five, five and four annotated genes. The annotated genes of inhibitor interval in different genomes showed high collinearity. **d** The scaffolds of 5V#4S and 5V#5S containing the homologs of annotated genes in the inhibitor interval of different reference genomes. The 5V#5S scaffold Scaffold4218 includes three annotated genes and 5V#4S scaffold Scaffold15749 includes two annotated genes; both them contain G3 alleles. Both 5V#5S scaffold Scaffold3806 and 5V#4S scaffold Scaffold38916 include G5 and G6 alleles, whereas, 5V#4S scaffold Scaffold15749 and 5V#5S scaffold Scaffold3806 share a high identity DNA fragment (2,166/2,120, 94.82%). Thus, the four scaffolds were assembled a contiguous 100,963 bp sequence between markers *SCA4218* and *SCA39816,* covering the inhibitor interval. This sequence was annotated six genes including an intact coiled-coil nucleotide-binding leucine-rich repeat (CNL) gene, *CNL1* (G4). G3 and G4 alleles were all absent on 5VS of 91C43^{DH}.

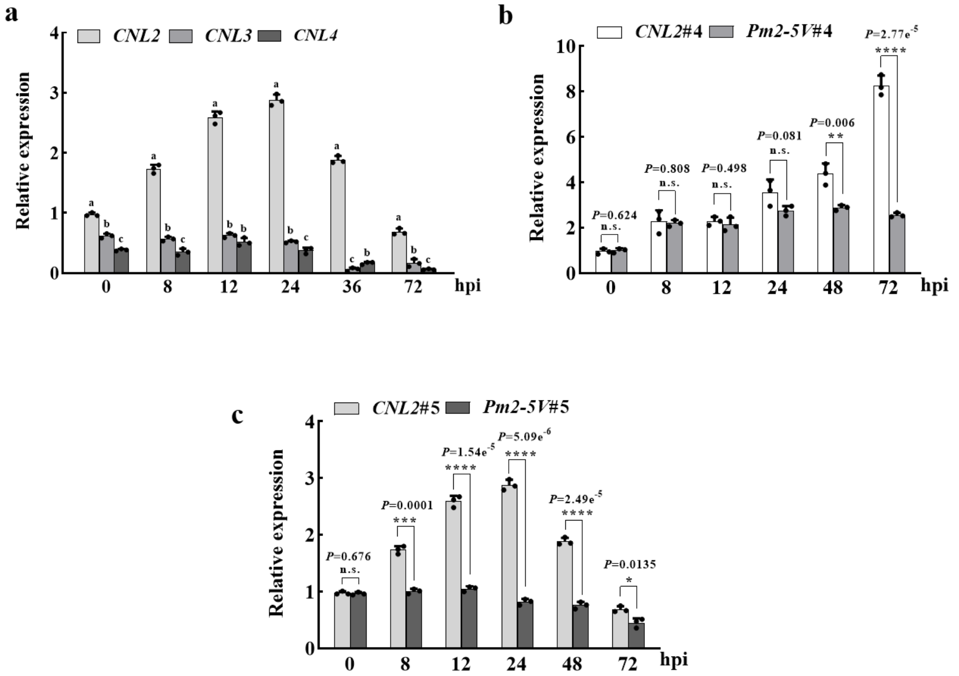

Supplementary Fig. 6. The expression analysis of *CNL2* alleles.

a The expression analysis of *CNL2*, *CNL3* and *CNL4* in NAU1908. *CNL2* showed significant up expression after inoculation with *Bgt* isolate E09, whereas *CNL3* and *CNL4* showed lower expression, indicating that *CNL2* could be the functional gene. Values are the mean ± SD of qRT-PCR (one-way ANOVA with Duncan, n = 3 biologically independent experiments). **b** Expression levels of *CNL2#4* and *Pm2-5V#4* in R5VS-15 inoculated with *Bgt* isolate E09. *CNL2#4* transcript levels were approximately eight-fold higher at 72 hpi. Values are the mean ± SD of qRT-PCR (two-sided *t*-test, n = 3 biologically independent experiments, ****P < 0.0001, **P < 0.01, N.S., no significant). **c** Expression levels of *CNL2#5* and *Pm2-5V#5* in NAU1908. *CNL2#5* transcript levels were approximately three-fold higher at 24 hpi. Values are the mean ± SD of qRT-PCR (two-sided *t*-test, n = 3 biologically independent experiments, ****P < 0.0001, ***P < 0.001, *P < 0.05, N.S., no significant). Source data are provided as a Source Data file.

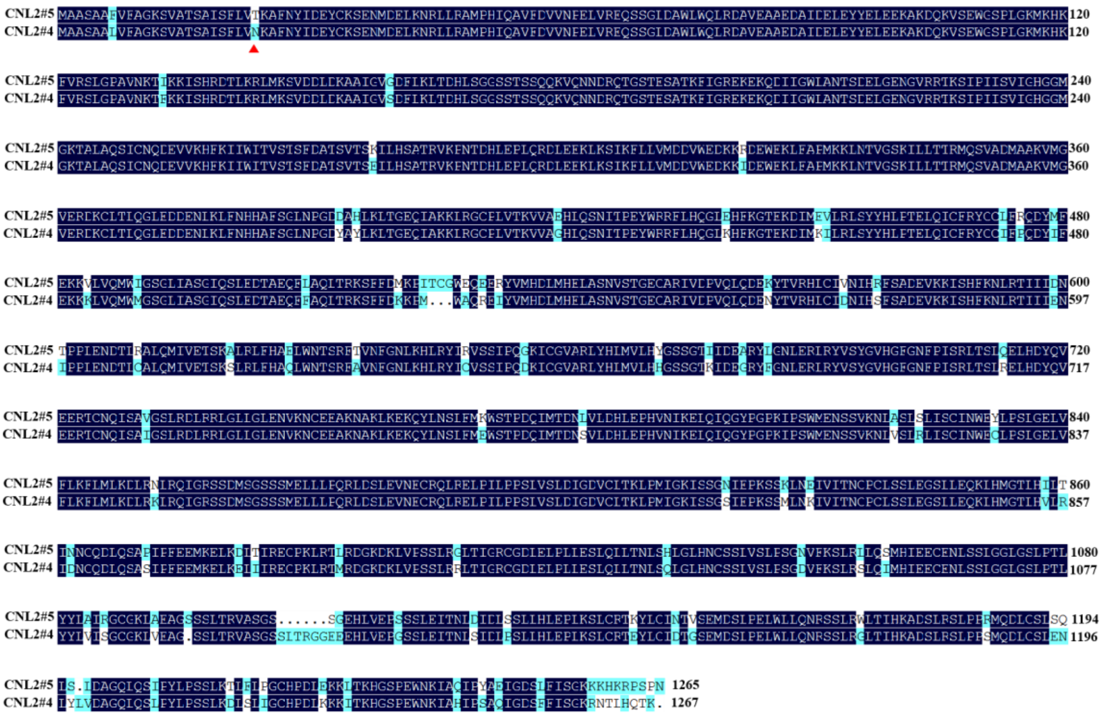

Supplementary Fig. 7. Protein sequences alignment between *CNL2#5* and *CNL2#4*.

CNL2#5 encodes 1,265 amino acids. *CNL2#4* encodes 1,267 amino acids. They share 91.5% identity and display a high level of sequence diversity in LRR domains, but only an amino acid (triangle indicating) in their CC domains (18 to 107) is difference.

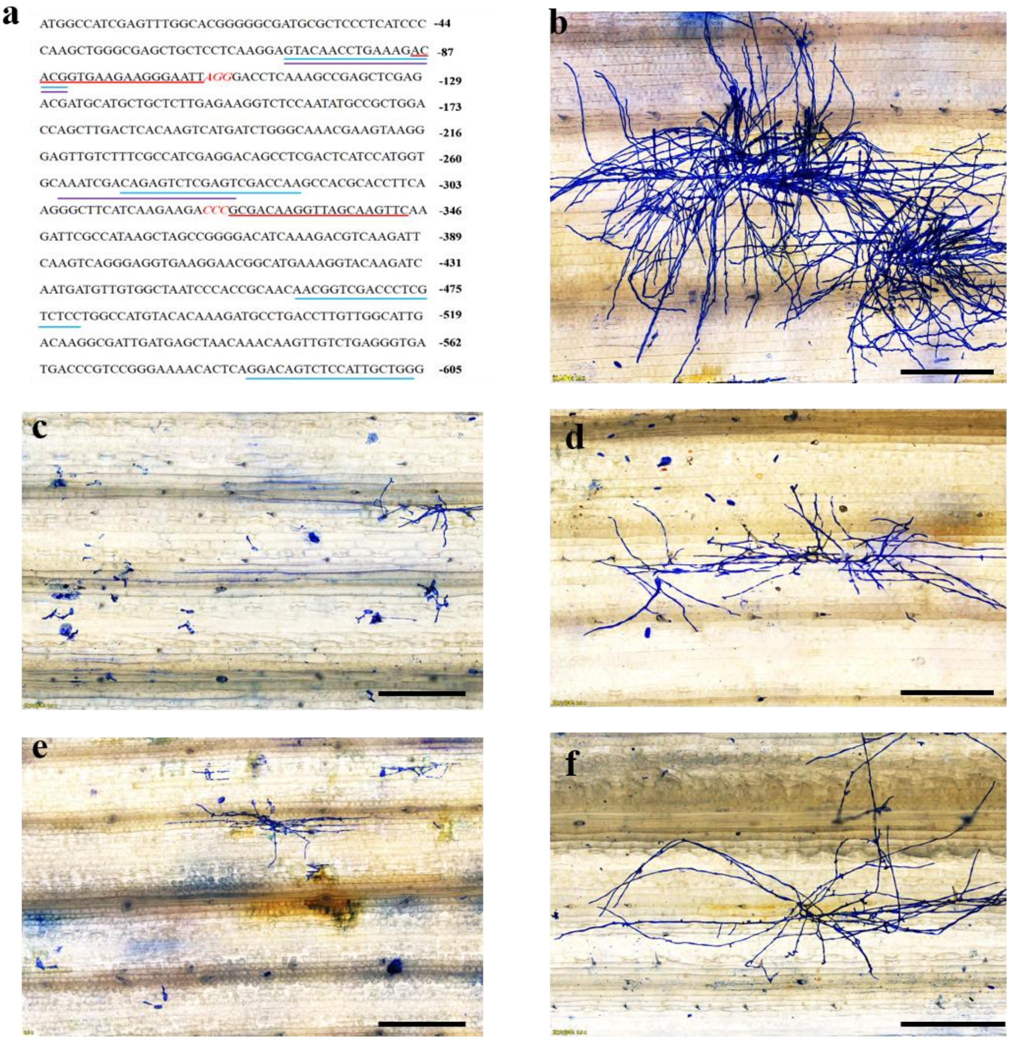

Supplementary Fig. 8. Microscopic observations of Coomassie brilliant blue staining at 72 hpi.

a The locations of the primers used in the gene expression analysis (blue lines indicating) of *SuPm55*, VIGS (purple lines indicating) and CRISPR/Cas9 (red lines indicating). **b-f** Microscopic observations of infected leaves from TF5V-1 (**b**), Del5VS-1(**c**), Del5VS-2 (**d**), Del5VS-3 (**e**), Del5VS-4 (**f**). The amounts of spores on the seedling leaves of four homozygous mutants significant reduced compared with TF5V-1. The micro-colony indexes of each line were showed in Fig. 2e. Bars, 200 um. Source data are provided as a Source Data file.

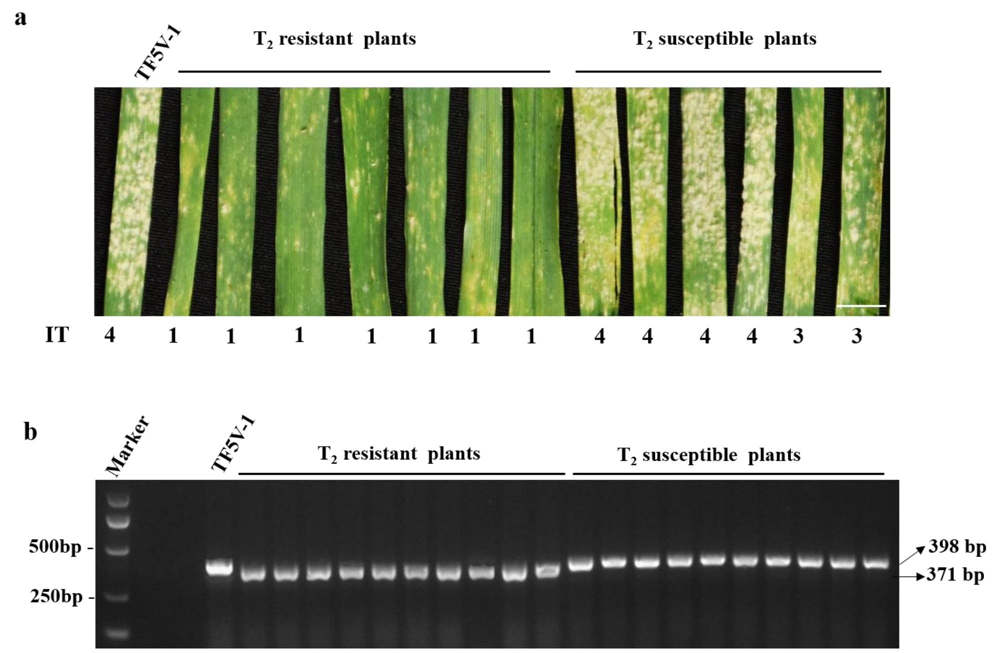

Supplementary Fig. 9. The response of Del5VS-4 T_2 transgenic plants to powdery mildew at seedling stage.

a Seedling responses to *Bgt* isolate E09 of the T_2 progeny derived from a Del5VS-4 T_1 heterozygote. Three independent repeats were performed. Bar = 0.5 cm. **b** PCR identification of T_2 plants showing that all resistant plants (IT 1) have the 27 bp deletion, whereas all susceptible plants (IT 3-4) have no deletion. The primers are F: TTAGGTACACGCAATTTCAA and R: TTGGTCGACTCGAGACTCTG.

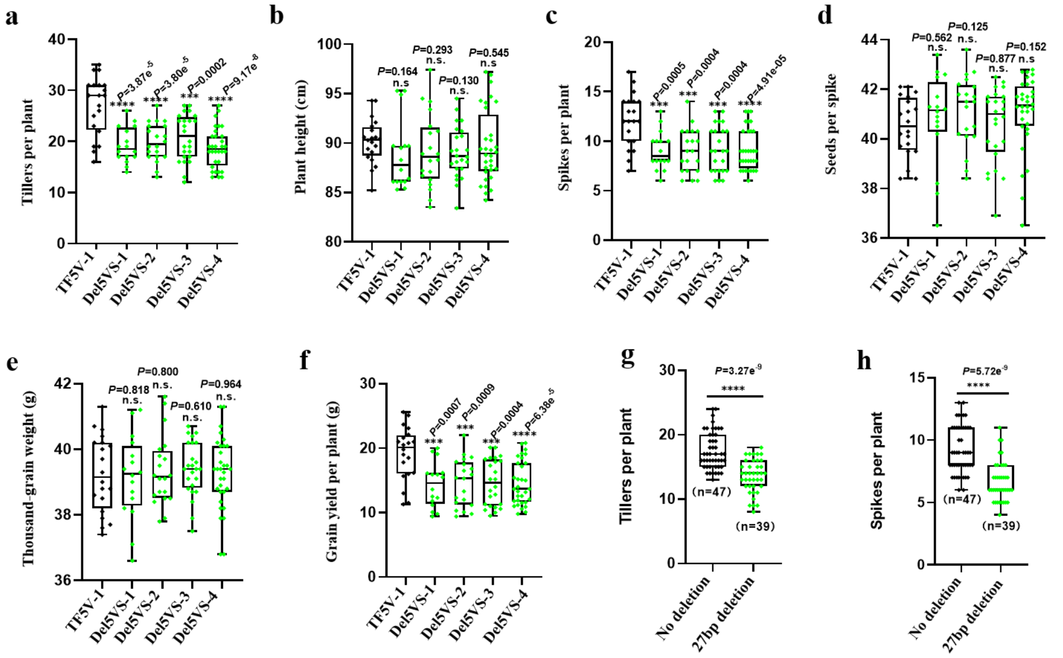

Supplementary Fig. 10. Agronomic traits of *SuPm55* knock out plants.

a-**f** The differences of the major traits in tiller number (**a**), plant height (**b**), spikes per plant **(c)**, seeds per spike (**d**), thousand-grain weight (**e**), grain yield per plant (**f**) between TF5V-1 and the *SuPm55* knock out lines. Values are the mean ± SD (two-sided *t*-test, ****$P < 0.0001$, ***$P < 0.001$, N.S., no significant), n=20 (TF5V-1), n=16 (Del5VS-1), n=20 (Del5VS-2), n=24 (Del5VS-3), n=32 (Del5VS-4). **g** Tiller number between none deletion plants (n=47) and 27 bp deletion plants (n=39). Values are the mean ± SD (two-sided *t*-test, ****$P < 0.0001$). **h** Spikes per plant between none deletion plants (n=47) and 27 bp deletion plants (n=39). Values are the mean ± SD (two-sided *t*-test, ****$P < 0.0001$). Source data are provided as a Source Data file.

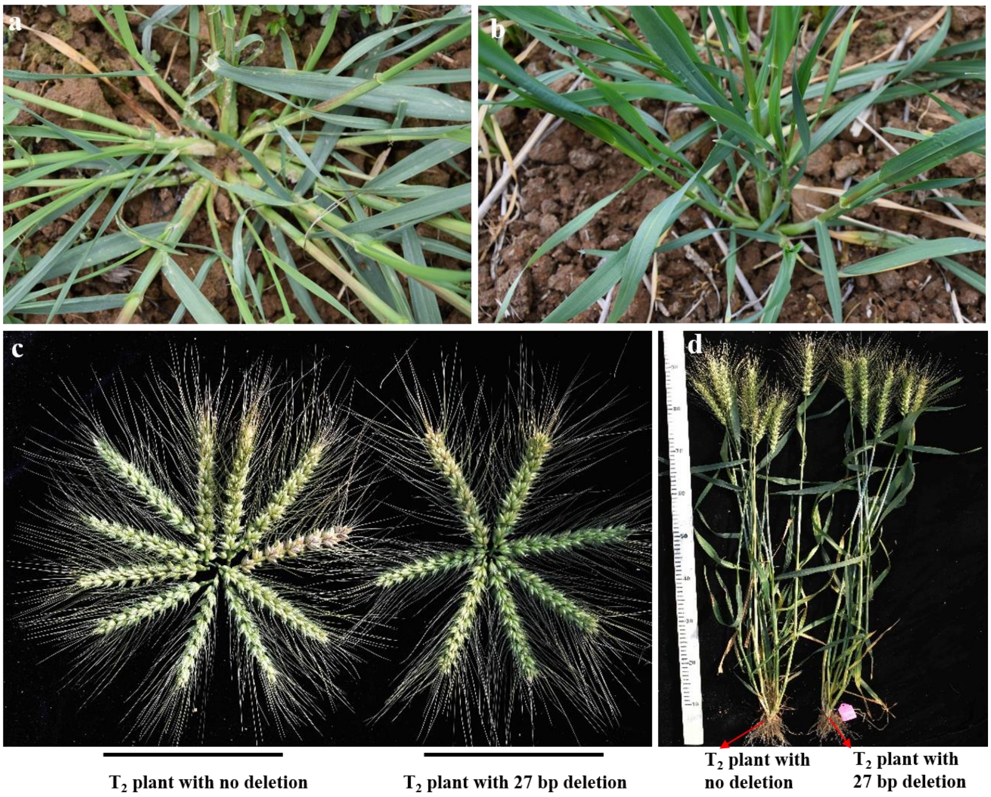

Supplementary Fig. 11. The morphologies of T_2 adult plants with or without the 27 bp deletion derived from a Del5VS-4 T_1 heterozygote.

a The T_2 plant with no deletion of *SuPm55* showing susceptible to powdery mildew on the leaf sheaths. **b** Homozygote plant with 27 bp deletion of *SuPm55* has no powdery mildew symptoms on all tissues, but significant lower in tiller number. **c** Homozygote plant with 27 bp deletion of *SuPm55* has lower spikes than that of the plant without 27 bp deletion. **d** Comparison of the height of plants with and without the 27 bp deletion.

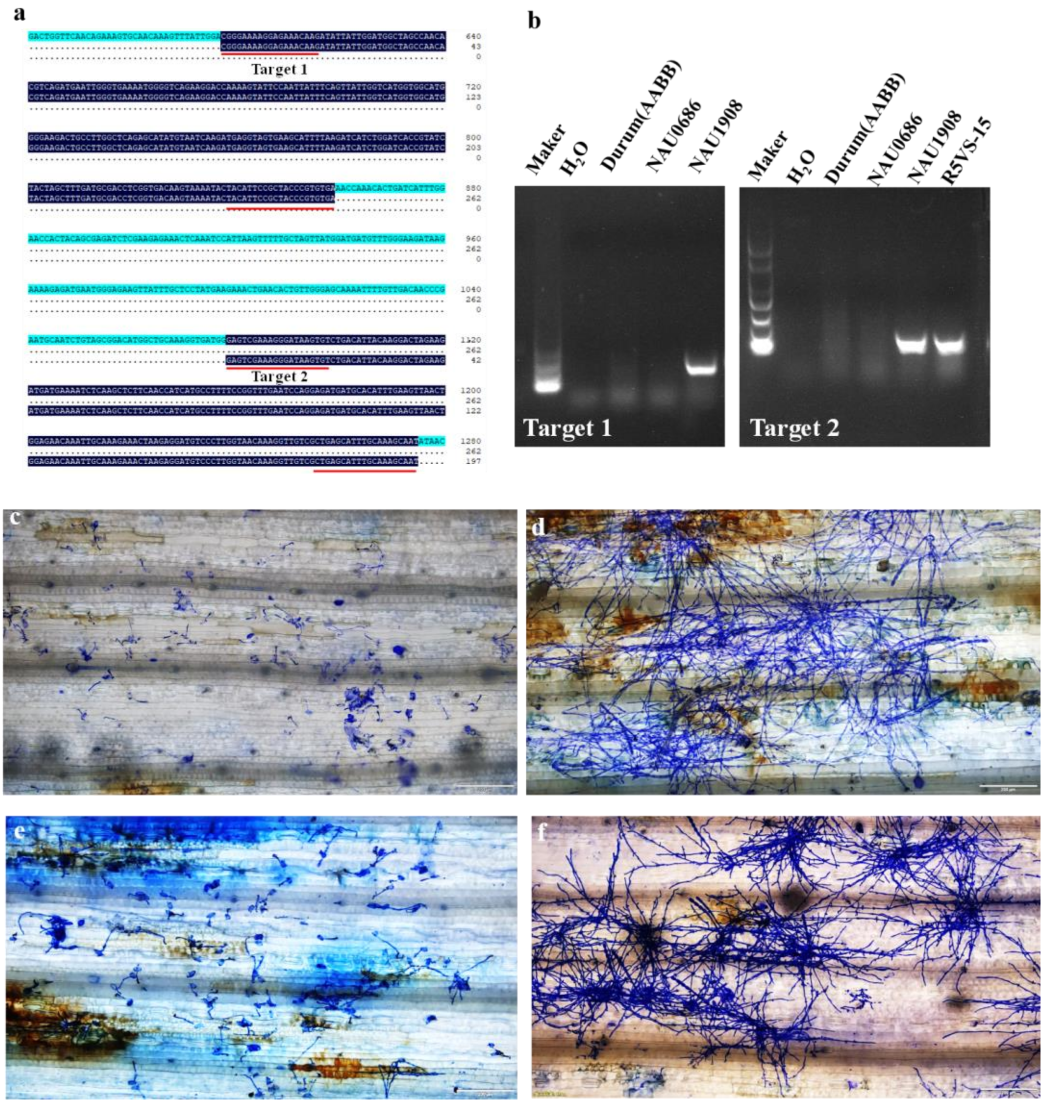

Supplementary Fig. 12. Microscopic observations of Coomassie brilliant blue staining displaying the presence of fungal structures at 72 h post-inoculation of VIGS leaves.

a The VIGS specific primers locations of target 1 used for CNL2#5, and target 2 used for CNL2#4 and CNL2#5 simultaneously (red lines indicating). **b** The specificity of primers PCR productions detected by gel electrophoresis. **c**-**f** Microscopic observations of BSMV: γ-infected leaf of NAU1908 (**c**), BSMV: *CNL2#5*-infected leaf of NAU1908 (**d**), BSMV: γ-infected leaf of R5VS-15 (**e**), and BSMV: *CNL2#4*-infected leaf of R5VS-15 (**f**). *Bgt* colonies are absence on the BSMV: γ-infected leaves of NAU1908 and TF5V-1, but present on the BSMV: *CNL2#5*-infected leaf of NAU1908 and BSMV: *CNL2#4*-infected leaf of R5VS-15. Bars, 200 μm.

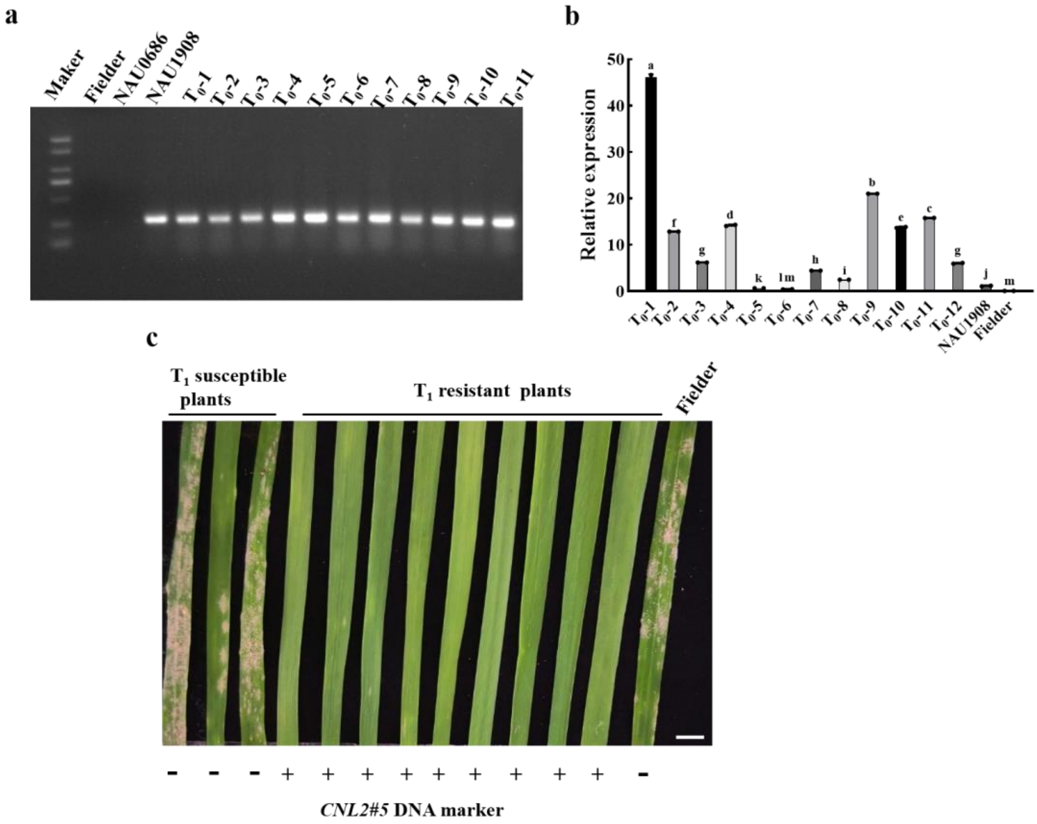

Supplementary Fig. 13. Molecular analysis of *CNL2#5* transgenic wheat.

a T_0 plants tested for presence of the transgene by PCR amplification with the *CNL2#5*-specific marker. **b** qRT-PCR analysis of *CNL2#5* expression in T_0 transgenic wheat lines. The values of qRT-PCR are the mean ± SD (two-sided *t*-test, n = 2 biologically independent experiments). **c** T_1 transgenic plants with or without *CNL2#5* response to *Bgt* isolate E09. Plants with *CNL2#5* are resistant to isolate E09, whereas those without *CNL2#5* are susceptible. Three independent repeats were performed. Presence (+) or absence (–) of the *CNL2#5* is indicated. Fielder was the negative control. Bar, 0.5 cm. Source data are provided as a Source Data file.

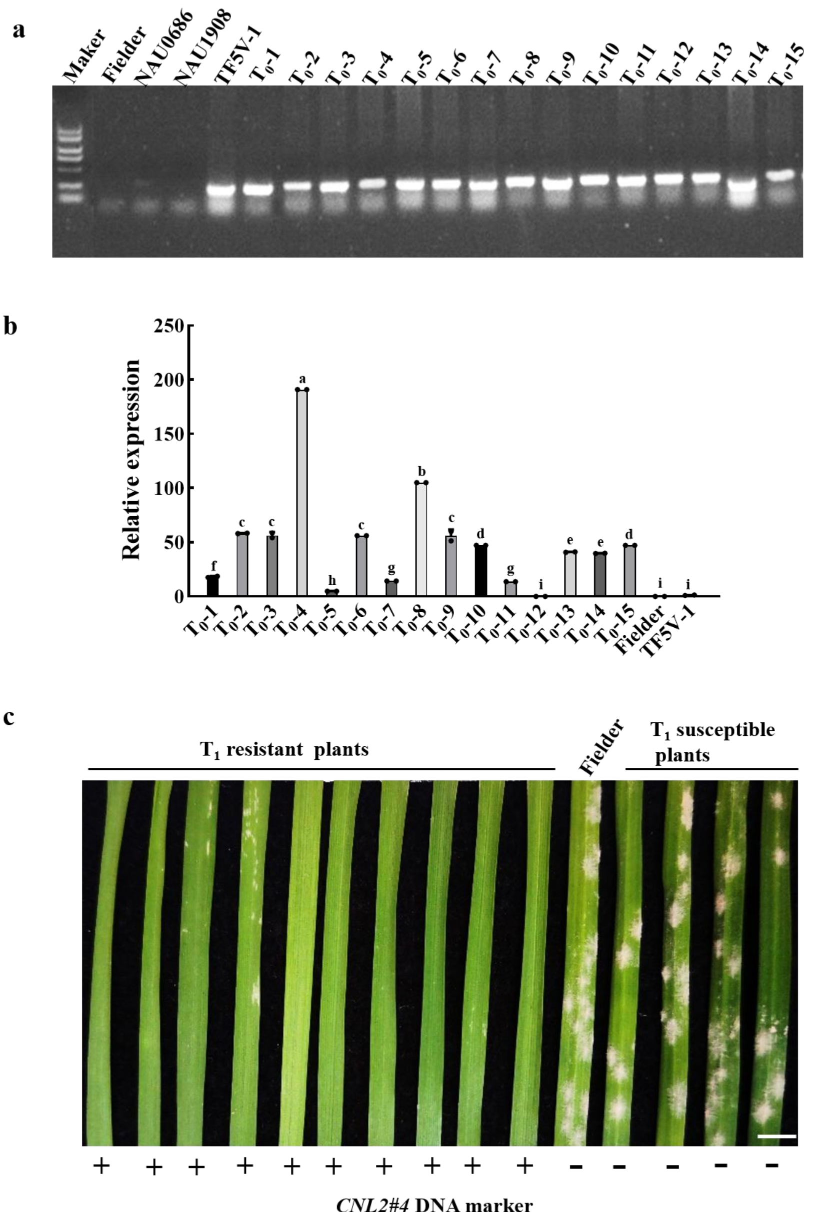

Supplementary Fig. 14. Molecular analysis of *CNL2#4* transgenic wheat.

a T_0 plants were tested for the presence of transgene by PCR amplification with the *CNL2#4*-specific marker. **b** qRT-PCR analysis of the expression of *CNL2#4* in T_0 transgenic wheat lines. The values of qRT-PCR are the mean ± SD (two-sided *t*-test, n = 2 biologically independent experiments). **c** T_1 transgenic plants with or without *CNL2#4* response to *Bgt* isolate E09. T_1 transgenic plants with *CNL2#4* are resistant to isolate E09, whereas those without *CNL2#4* are susceptible. Three independent repeats were performed. Presence (+) or absence (–) of *CNL2#4* is indicated. Fielder was the negative control. Bar, 0.5 cm. Source data are provided as a Source Data file.

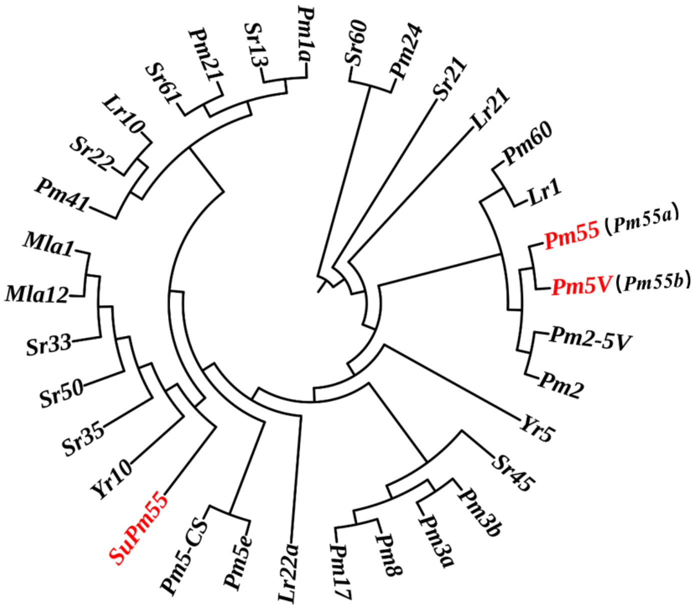

Supplementary Fig. 15. Phylogenetic tree of *Pm55* and *SuPm55* proteins.

Reported R proteins were selected from wheat, barley and rye for building the neighbor-joining tree; *Lr1* (ABS29034.1), *Lr21* (ACO5339 7.1), *Lr22a* (ARO38245.1), *Pm2* (CZT14023.1), *Pm3b* (AAQ96158.1), *Sr22* (CUM44200.1), *Sr45* (CUM44213.1), *Yr5* (QEQ12705.1), *Yr10* (AAG42167.1), *Sr13* (ATE88995.1), *Sr60* (MK629715.1) *Pm60* (AUO29720.1), *Sr21* (AVK42834.1), *Sr35* (AGP75918.1), *Sr33* (AGQ17382.1), *Pm8* (AGY30894.1), *Sr50* (ALO61074.1), *Pm21* (AVR59184.1), *Mla1* (AAG37356.1), *Mla12* (AAO43441.1), *Pm1a* (MT773601.1), *Pm3a* (AY939880.1), *Pm5e* (MK955156.1), *Pm5-CS* (MK955157.1), *Pm17* (MH077963.1), *Pm24* (MK950855.1), *Pm41* (MV395289.1), *Sr61* (MN531844.1) and *Lr10* (GU393304.1).

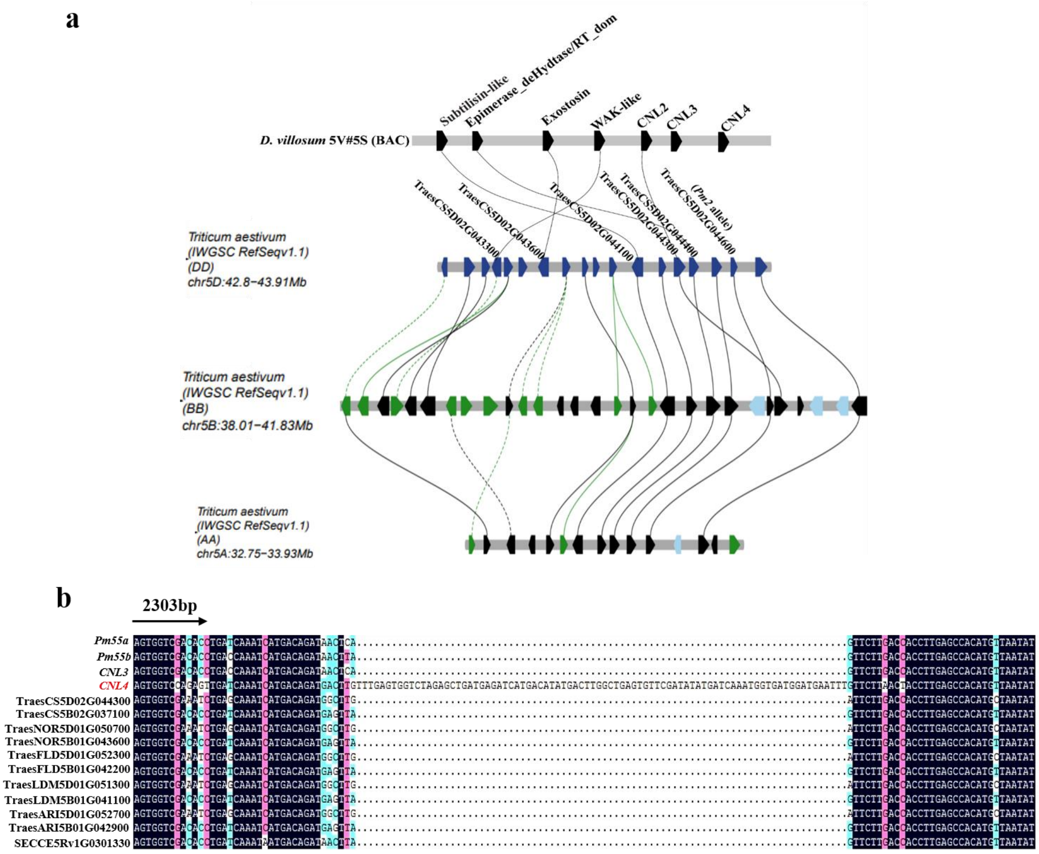

Supplementary Fig. 16. Comparison of the *PM55* loci from the bread wheat and relatives.

a Comparison of the *PM55* loci from Chinese Spring genomes reveals low sequence conservation and chromosomal rearrangement. **b** *Pm55*-like genes present in the wheat genomes of 5BS and 5DS and rye 5RS all lack an 84 bp sequence in *CNL4*, suggesting that *CNL4* is not orthologous to *Pm55*-like genes in the other genomes.

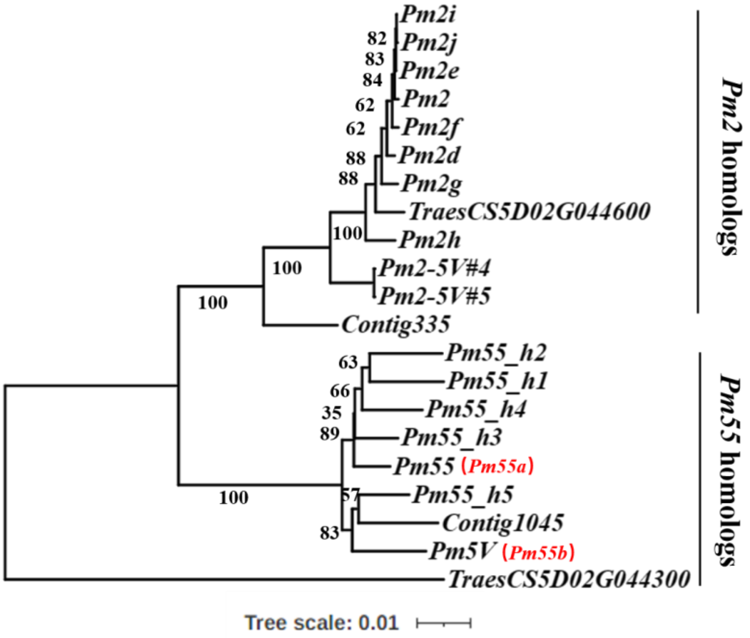

Supplementary Fig. 17. Phylogenetic relationships among *Pm2* and *Pm55* homologs in wheat and *D. villosum*.

Pm2i (MW538907.1), *Pm2j* (MW538906.1), *Pm2e* (MW538904.1), *Pm2* (CZT14023.1), *Pm2f* (MW538905.1), *Pm2d* (MW538903.1), *Pm2g* (MW538910.1), *Pm2h* (MW538911.1), *Pm2-5V#5*(OM646566.1), *Pm2-5V#4* (OM646567.1), Contig335 (MZ672471.1), Contig1045 (MZ672843.1) and TraesCS02G044600. Contig335 and Contig1045 are NLRs isolated from *D.villosum* through resistance gene enrichment sequencing (RenSeq).

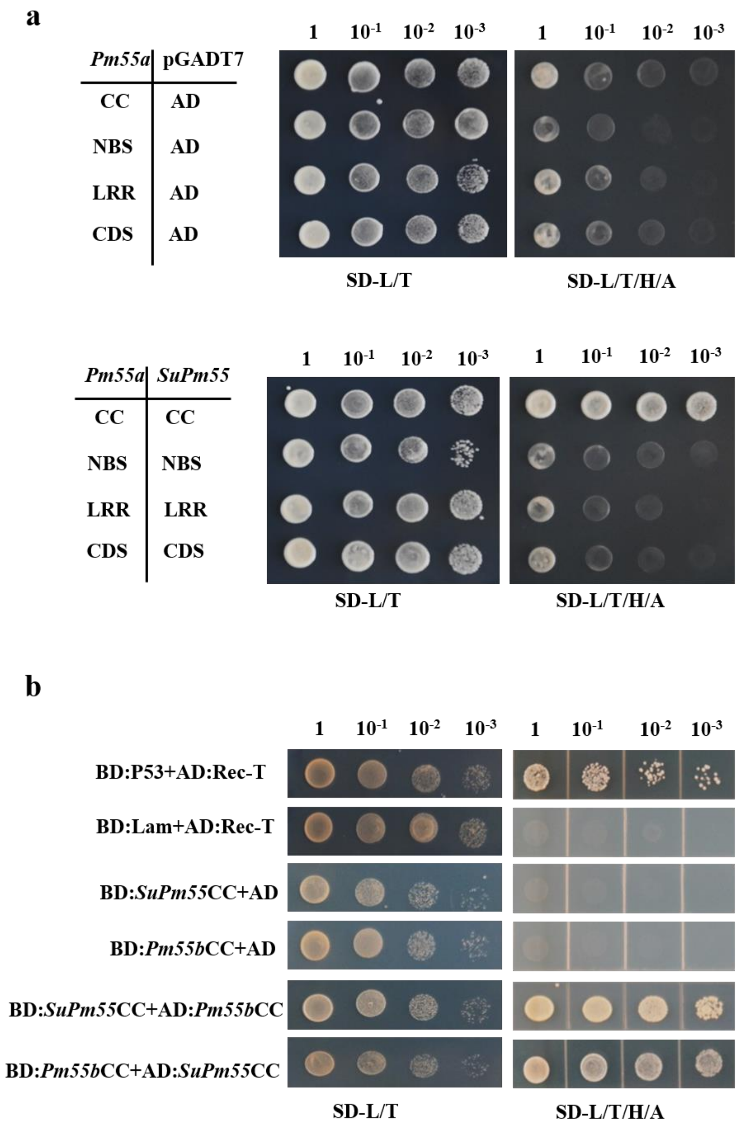

Supplementary Fig. 18. Yeast two-hybrid assay to identify protein interaction.

a Yeast two-hybrid assay confirmed that the CC domains of *Pm55a* and *SuPm55* are required for pairwise interaction of *Pm55a* and *SuPm55*, confirmed by growth of yeast cells on basal media without supplement of Trp, Leu, Ade and His. **b** Yeast two-hybrid assay confirmed that the CC domains of *Pm55b* and *SuPm55* interacted.

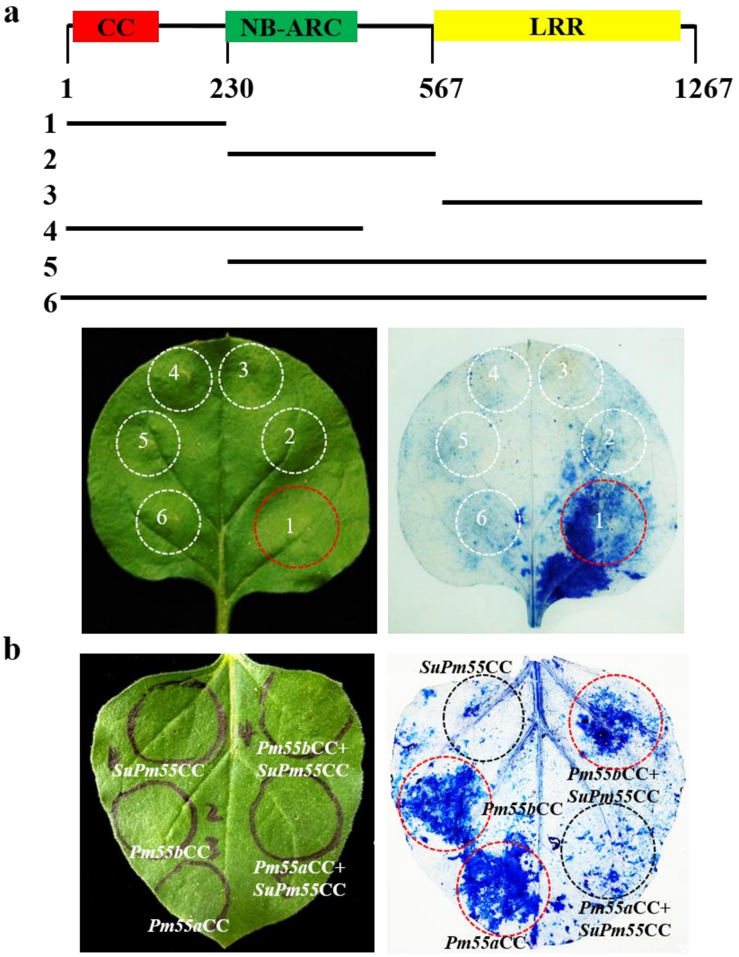

Supplementary Fig. 19. Cell death assay in *N. benthamiana* leaves.

a Cell death induction by *Pm55* domains when *N. benthamiana* leaves were infiltrated with *Agrobacteria* carrying different constructs. Cell death was observed by trypan blue staining at 48 h post infiltration. The assays showed that overexpression of the *Pm55* CC domain (**1**) alone in *N. benthamiana* leaves induced cell death in the injection region, whereas the longer CC-NBS (**4**) or CC-NBS-LRR (**6**) did not induce cell death responses. **b** The CC domain of *SuPm55* suppressed hypersensitive response cell death induced by *Pm55a* in tobacco leaves, but did not inhibit *Pm55b*-triggered cell death.

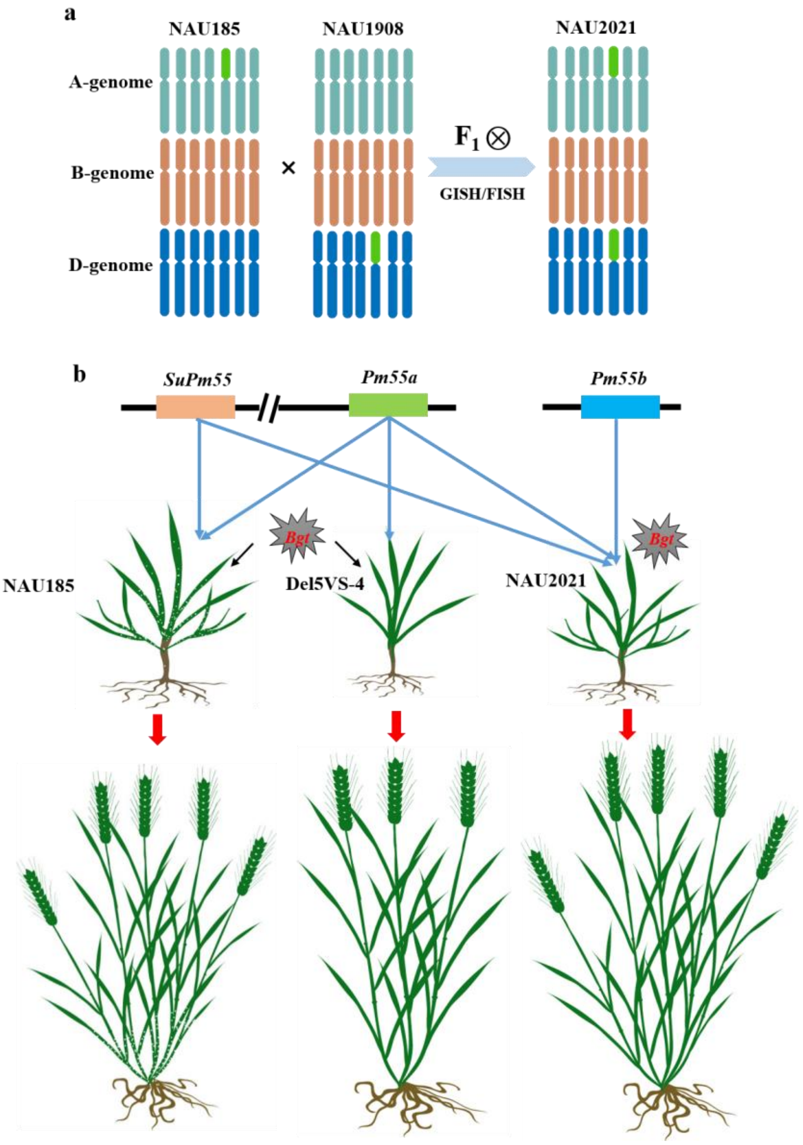

Supplementary Fig. 20. Pyramiding *SuPm55*/*Pm55a* and *Pm55b* in wheat.

a Homozygous multi-translocations line NAU2021 was identified by using GISH/FISH in the F_2 progeny of NAU185 (*SuPm55/Pm55a*)/NAU1908 (*Pm55b*). **b** The 5VS-translocation with *SuPm55*/*Pm55* in wheat exhibits adult-plant tissue-specific resistance, whereas that with *Pm5V* shows all-stage resistance. The inactivation of *SuPm55* significantly reduces grain yield, indicating that expression of *SuPm55* in both seedlings and adult-leaf sheaths contributes to enhancing the plant fitness. Combining *SuPm55*/*Pm55* and *Pm5V* in wheat through developing T5AL·5V#4S and T5DL·5V#5S translocations line NAU2021 shows no mutual allele suppression and yield penalty.

Supplementary Table 1. Plant materials used in the study.

Line	Chromosome structure	Description
91C43	VV#4 (2n=14)	*Dasypyrum villosum* accession introduced from British
01I140	VV#5 (2n=14)	*Dasypyrum villosum* accession collected in Italy
01I141	VV#6 (2n=14)	*Dasypyrum villosum* accession collected in Greece
01I142	VV#7 (2n=14)	*Dasypyrum villosum* accession collected in Greece
01I143	VV#8 (2n=14)	*Dasypyrum villosum* accession collected in Turkey
01I144	VV#9 (2n=14)	*Dasypyrum villosum* accession collected in Italy
01I145	VV#10 (2n=14)	*Dasypyrum villosum* accession collected in Bulgaria
NAU0686	AABBDD (2n=42)	Susceptible bread wheat cultivar used as the recurrent parent
NAU185	T5AL·5V#4S (2n=42)	Chromosome arm 5VS of 91C43 substituted 5AS of NAU0686
TF5V-1	T5DL·5V#4S (2n=42)	Chromosome arm 5VS of 91C43 substituted 5DS of NAU0686
NAU1908	T5DL·5V#5S (2n=42)	Chromosome arm 5VS of 01I140 substituted 5DS of NAU0686

Supplementary Table 2. Responses of F_1 and F_2 populations derived from cross R5VS-15/TF5V-1 when infected with *Bgt* isolate E09 at seedling stage.

Population	No. of the infected plants	No. of resistant plants	No. of susceptible plants	Expected ratio	χ^2	*P*-value
F_1	28	0	28	-	-	-
F_2	522	128	394	1:3	0.064	0.8

Supplementary Table 3. The responses to *Bgt* isolate E09 of a T_2 population derived from a Del5VS-4 T_1 heterozygote.

	Homozygous plants with 27 bp deletion	Heterozygous plants	Homozygous plants without deletion	Expected ratio	χ^2	*P*-value
No. plants	39	81	47	1:2:1	0.88	0.644
Seedling resistance to E09 (IT 0-4)	R (1-2)	S (3-4)	S (3-4)	1:3	0.07	0.791
Adult-plant resistance on leaves (IT 0-9)	R (0;-1)	R (0;-1)	R (0;-1)	-	-	-
Adult-plant resistance on sheathes (IT 0-9)	R (1-2)	S (6-8)	S (7-8)	1:3	0.07	0.791

Supplementary Table 4. Segregation of powdery mildew response in T_1 families of *CNL2* alleles.

**Line**	**No. of the infected plants**	**No. of resistant plants**	**No. of susceptible plants**	**Expected ratio**	χ^2	***P*-value**
ProCNL2#5:CNL2#5T_0-9	63	56	7	3:1	6.48	0.011
ProCNL2#5:CNL2#5T_0-10	54	49	5	3:1	7.13	0.008
ProCNL2#5:CNL2#5T_0-11	82	72	10	3:1	7.17	0.007
ProCNL2#4:CNL2#4T_0-2	59	41	18	3:1	0.95	0.33
ProCNL2#4:CNL2#4T_0-3	107	78	29	3:1	0.25	0.617
ProCNL2#4:CNL2#4T_0-4	71	65	6	3:1	10.36	0.001

Supplementary Table 5. Seedling infection types (IT 0-4) produced by 18 *Bgt* isolates.

Isolate	Fielder	R5VS-15（*Pm55a*）	NAU1908（*Pm55b*）	R5VS-27（*Pm55b*+ *SuPm55*）	NAU2021（*SuPm55/Pm55a* + *Pm55b*）	CNL2#4T2-3（*Pm55a*）	CNL2#5T2-9（*Pm55b*）
HB-24(SWQ)	4	0	0	0	0	0	0
HB-24	4	1	0	0	0	2	0
14-32	4	0;	0	0	0	0;	0
1-19	4	0	0	0	0	0	0
21-2	4	1	0;	0;	0	2	1
11-99	4	0	0	0	0	0	0
96224	4	0;	0;	0;	0	0;	0;
46-30	4	1	0	0	0	1	0
11-133	4	0	0	0	0	0	0
15-17	4	0	0	0	0	0	0
12-3	4	0;	0	0	0	0;	0
46-25	4	0;	0	0;	0	0;	0
48-18	4	2	2	1	0	2	2
48-28	4	3	2	2	2	4	2
NJ-16	4	3	2	2	2	4	2
E21-4	4	3	0	0	0	4	0
HG-1	4	2	0	0	0	2	0
39-19	4	1	0	0	0	1	0

Supplementary Table 6. Mutations of *CNL2* alleles in susceptible NAU1908 and TF5V-1 mutants.

Gene	M_1 plant	Mutation in cDNA	Amino acid change	Motif or domain
CNL2#5	M18	C2392T	L798Stop	LRR
CNL2#5	M477	G3194A	C1065Y	LRR
CNL2#5	M512	C3266T	P1089L	LRR
CNL2#4	PmS5	C1532T	A511V	Linker between NBS and LRR
CNL2#4	PmS21	T320G	V107G	CC domain
CNL2#4	PmS27	G2129A	R710Q	LRR
CNL2#4	PmS29	G259A	E87K	CC domain
CNL2#4	PmS32	G1342A	D448N	NB-ARC
CNL2#4	PmS34	G1621A	A541T	Linker between NBS and LRR
CNL2#4	PmS37	C1952T	P651L	LRR
CNL2#4	PmS39	G2130A	R710G	LRR
CNL2#4	PmS40	T3266C	V1089A	LRR
CNL2#4	PmS41	G3095A	G1032E	LRR

Supplementary Table 7. Orthologs of *Pm55* and *Pm2* on homoeologous chromosomes of bread wheat and its relatives.

Accession	Homoeologous chromosomes	Homologous *PM55* locus	*Pm55* identity (%)	Homologous *PM2* locus	*Pm2* identity (%)
Chinese Spring (AABBDD)	5DS	TraesCS5D02G044300	82.5	TraesCS5D02G044600	99.5
	5BS	TraesCS5B02G037100	91.4	TraesCS5B02G037000	96.5
	5AS	——		TraesCS5A02G036400	95.7
Norin61 (AABBDD)	5DS	TraesNOR5D01G050700	82.5	TraesNOR5D01G051000	99.5
	5BS	TraesNOR5B01G043600	91.4	TraesNOR5B01G043500	96.5
	5AS	——		TraesNOR5A01G042200	95.7
Fielder (AABBDD)	5DS	TraesFLD5D01G052300	82.5	TraesFLD5D01G052600	99.5
	5BS	TraesFLD5B01G042200	91.4	TraesFLD5B01G042100	97.9
	5AS	——		TraesFLD5A01G041600	96.2
Land mark (AABBDD)	5DS	TraesLDM5D01G051300	82.5	TraesLDM5D01G051600	99.5
	5BS	TraesLDM5B01G041100	91.4	TraesLDM5B01G041000	97.9
	5AS	——		TraesLDM5A01G048200	96.2
Arinalr For (AABBDD)	5DS	TraesARI5D01G052700	82.5	TraesARI5D01G053000	99.5
	5BS	TraesARI5B01G042900	91.5	TraesARI5B01G042800	93.8
	5AS	——	-	TraesARI5A01G040400	95.7
T. urartu G1812 (AA)	5AS	——	-	TuG1812G0500000446	95.7
Rye Lo7 (RR)	5RS	SECCE5Rv1G0301330	91.0	SECCE5Rv1G0301340	94.3
NAU1908	5V#5S	*Pm5V*（*Pm55b*）	95.2	*Pm2-5V#5*	95.3
TF5V-1	5V#4S	*Pm55* （*Pm55a*）	100	*Pm2-5V#4*	94.5

Supplementary Table 8. DNA sequence identify of *Pm55* haplotypes in *D. villosum*.

Accession	Haplotype	***Pm55***	***Pm5V***	***Pm55_h1***	***Pm55_h2***	***Pm55_h3***	***Pm55_h4***	***Pm55_h5***
91C43	*Pm55*	100						
01I140	*Pm5V*	95.2	100					
01I141	*Pm55_h1*	96.1	96.0	100				
01I142	*Pm55_h2*	95.7	93.1	93.6	100			
01I143	*Pm55_h3*	96.9	96.6	97.0	94.0	100		
01I144	*Pm55_h4*	96.4	96.4	96.9	93.6	97.1	100	
01I145	*Pm55_h5*	96.1	93.7	92.9	95.5	94.1	95.3	100

Supplementary Table 9. Average rate of nucleotide substitutions per 100 sites among *Pm55* alleles/haplotypes.

Rate	Complete gene	CC	NB-ARC	LRR
Ka	0.03	0.01	0.01	0.06
Ks	0.05	0.06	0.01	0.08
Ka/Ks	0.6	0.16	1.00	0.75

Supplementary Table 10. Primers used for cloning and functional analysis.

Primer	Sequence (5′–>3′)	Amplified size (bp)	Utilization
Pm55/Pm5V gDNA	F: GATCCTGACCATGGAAATTTC<br>R: TTCTATGCGCCAAACCTTGTTC	-	*Pm55* alleles gDNA cloning
Pm55/Pm5V cDNA	F: ATGGCTGCCTCTGCTGCATTTGA<br>R: TTAGTTTGGTGAAGGCCTTTTG	-	*Pm55* alleles cDNA cloning
Pm55/Pm5V promoter	F: AAGAGCCCATAATCATGGTCAC<br>R: GCAGTACTCATCGATGTAGT	-	*Pm55* alleles promotor cloning
Pm55/Pm5V terminator	F: GCTGACTATACACAAAGCAGA<br>R: CTGACCTGAACATATGTTCG	-	*Pm55* alleles 3'UTR cloning
Pm5V VIGST$_1$	F: CATTTTTTTTTTTTTTTAGCTAG<br>CCGGGAAAAGGAGAAACAAGAT<br>R: GATGATTCTTCTTCCGTTGCTA<br>GCTCACACGGGTAGCGGAATGT	262	For *Pm55b* VIGS vector construction (target 1)
Pm5V VIGST$_2$	F: CATTTTTTTTTTTTTTTAGCTA<br>GCGAGTCGAGAGGGATAAGTGT<br>R: GATGATTCTTCTTCCGTTGCT<br>AGCATTGCTTTGCAAATGCCCA	197	For *Pm55b* VIGS vector construction (target 2)
Pm5V qPCRT1	F: GAGTCGAGAGGGATAAGTGT<br>R: ATTGCTTTGCAAATGCCCA	229	*Pm55b* expression analysis (target 1)
Pm5V qPCRT2	F: CGGGAAAAGGAGAAACAAGAT<br>R: TCACACGGGTAGCGGAATGT	197	*Pm55b* expression analysis (target 2)
Pm55 VIGS	F: CATTTTTTTTTTTTTTTAGCTA<br>GCGAGTCGAGAGGGATAAGTGT<br>R: GATGATTCTTCTTCCGTTGCT<br>AGCATTGCTTTGCAAATGCCCA	197	*Pm55a* VIGS vector construction
Pm55 qPCR	F: CGGGAAAAGGAGAAACAAGAT<br>R: TCACACGGGTAGCGGAATGT	195	*Pm55a* expression analysis
SuPm55 gDNA	F: TTAGGTACACGCAATTTCAA<br>R: CCTCAAATTAAGCTAGTGAG	2,749	*SuPm55* gene cloning
SuPm55 VIGS	F: CATTTTTTTTTTTTTTTAGCTA<br>GCGTACAACCTGAAAGACACG<br>R: GATGATTCTTCTTCCGTTGCT<br>AGCACTCGAGACTCTGTCGATTT	211	*SuPm55* VIGS vector construction
SuPm55 qPCRT1	F: GTACAACCTGAAAGACACG<br>R: TTGGTCGACTCGAGACTCTG	218	*SuPm55* expression analysis
SuPm55 qPCRT2	F: AACGGTCGACCCTCGTCTCC<br>R: CCAGCAATGGAGACTGTCC	144	*SuPm55* expression analysis
Pm2-5V gDNA	F: ATAACCCTTCCCATGCCTCT<br>R: AGTTTACTGCACTCGGGGAT	-	*Pm2-5V* homologous gene cloning
Pm2-5V qPCR	F: TCGTCACTAAGAGAACTAG<br>R: GTGAGTTTACTGCACTTGGAG	265	*Pm2-5V* expression analysis
