# Supplementary material for: Wheat Pm55 alleles exhibit distinct interactions with an inhibitor to cause different powdery mildew resistance
Source: Nat Commun. 2024 Jan 13;15:503. doi: 10.1038/s41467-024-44796-0 (PMC10787760; doi:10.1038/s41467-024-44796-0)
Supplement: Supplementary file 3 — Description of Additional Supplementary Files [file 41467_2024_44796_MOESM3_ESM.pdf]

Description of Additional Supplementary Files

File name: Supplementary Data 1

Description: The detailed information of molecular markers information used in Supplementary Fig 2.

File name: Supplementary Data 2

Description: The detailed information of the five molecular markers (SCA428, SCA3806, SCA15749, SCA39816 and Xdv-14) used in Supplementary Fig. 4.
