# Supplementary material for: Wheat Pm55 alleles exhibit distinct interactions with an inhibitor to cause different powdery mildew resistance
Source: Nat Commun. 2024 Jan 13;15:503. doi: 10.1038/s41467-024-44796-0 (PMC10787760; doi:10.1038/s41467-024-44796-0)
Supplement: Supplementary file 6 — Reporting Summary [file 41467_2024_44796_MOESM6_ESM.pdf]

nature portfolio

Corresponding author(s): Ruiqi Zhang

Last updated by author(s): Dec 16, 2023

Reporting Summary

Nature Portfolio wishes to improve the reproducibility of the work that we publish. This form provides structure for consistency and transparency in reporting. For further information on Nature Portfolio policies, see our Editorial Policies and the Editorial Policy Checklist.

Statistics

For all statistical analyses, confirm that the following items are present in the figure legend, table legend, main text, or Methods section.

n/a	Confirmed	
☐	☒	The exact sample size (*n*) for each experimental group/condition, given as a discrete number and unit of measurement
☐	☒	A statement on whether measurements were taken from distinct samples or whether the same sample was measured repeatedly
☐	☒	The statistical test(s) used AND whether they are one- or two-sided<br>*Only common tests should be described solely by name; describe more complex techniques in the Methods section.*
☒	☐	A description of all covariates tested
☐	☒	A description of any assumptions or corrections, such as tests of normality and adjustment for multiple comparisons
☐	☒	A full description of the statistical parameters including central tendency (e.g. means) or other basic estimates (e.g. regression coefficient) AND variation (e.g. standard deviation) or associated estimates of uncertainty (e.g. confidence intervals)
☐	☒	For null hypothesis testing, the test statistic (e.g. *F*, *t*, *r*) with confidence intervals, effect sizes, degrees of freedom and *P* value noted<br>*Give P values as exact values whenever suitable.*
☒	☐	For Bayesian analysis, information on the choice of priors and Markov chain Monte Carlo settings
☒	☐	For hierarchical and complex designs, identification of the appropriate level for tests and full reporting of outcomes
☒	☐	Estimates of effect sizes (e.g. Cohen's *d*, Pearson's *r*), indicating how they were calculated

Our web collection on statistics for biologists contains articles on many of the points above.

Software and code

Policy information about availability of computer code

Data collection	Confocal microscopic images were aquired with a Leica SP8 confocal microscopy.<br>LightCycler® 480 for qPCR data collection and analysis.<br>NightShade LB985(Berthold Technologies, Germany) for fluorescence detection.
Data analysis	1. MEGA 6.0 software<br>2. Microsoft excel<br>3. GraphPad Prism 8<br>4. SPSS 26.0 software<br>5. IndiGO software<br>6. DNAMAN 7.0<br>7. SMART(http://smart.embl-heidelberg.de/)

For manuscripts utilizing custom algorithms or software that are central to the research but not yet described in published literature, software must be made available to editors and reviewers. We strongly encourage code deposition in a community repository (e.g. GitHub). See the Nature Portfolio guidelines for submitting code & software for further information.

Data

Policy information about [availability of data](#)

All manuscripts must include a data availability statement. This statement should provide the following information, where applicable:

- Accession codes, unique identifiers, or web links for publicly available datasets
- A description of any restrictions on data availability
- For clinical datasets or third party data, please ensure that the statement adheres to our policy

Data supporting the findings of this work are available within the paper and Supplementary Information files. The plant materials and datasets generated and analyzed during the present study are available from the corresponding authors upon request. Detailed genomic sequences of Pm55 (OQ928403), Pm5V (ON109832), SuPm5V (OQ928410), Pm2-5V#4 (OQ928409), Pm2-5V#5 (OM646566) and Pm55_h1 to Pm55_h5 (OQ928404 to OQ928408) were deposited in NCBI Genbank. The following public databases were used in this study: D. villosum 91C43DH genome (https://bigd.big.ac.cn/), IWGSC RefSeq v2.1 (https://wheat-urgi.versailles.inra.fr/Seq), and Triticeae genomes (http://wheatomics.sdau.edu.cn/). Source data are provided as a Source data file.

Research involving human participants, their data, or biological material

Policy information about studies with human participants or human data. See also policy information about sex, gender (identity/presentation), and sexual orientation and race, ethnicity and racism.

Reporting on sex and gender	n/a
Reporting on race, ethnicity, or other socially relevant groupings	n/a
Population characteristics	n/a
Recruitment	n/a
Ethics oversight	n/a

Note that full information on the approval of the study protocol must also be provided in the manuscript.

Field-specific reporting

Please select the one below that is the best fit for your research. If you are not sure, read the appropriate sections before making your selection.

☒ Life sciences ☐ Behavioural & social sciences ☐ Ecological, evolutionary & environmental sciences

For a reference copy of the document with all sections, see nature.com/documents/nr-reporting-summary-flat.pdf

Life sciences study design

All studies must disclose on these points even when the disclosure is negative.

Sample size	Sample sizes were not predetermined, but chosen to be similar to common sample sizes in previous studies in the field.
Data exclusions	We did not exclude any data points arbitrarily.
Replication	All attempts at replication were successful. The number of replications is indicated in the corresponding figure legend.
Randomization	All the samples from each experiments was collected randomly for further analysis.
Blinding	All investigations were blinded to group allocation during data collection and analysis.

Reporting for specific materials, systems and methods

We require information from authors about some types of materials, experimental systems and methods used in many studies. Here, indicate whether each material, system or method listed is relevant to your study. If you are not sure if a list item applies to your research, read the appropriate section before selecting a response.

Materials & experimental systems

n/a	Involved in the study
☐	☒ Antibodies
☒	☐ Eukaryotic cell lines
☒	☐ Palaeontology and archaeology
☒	☐ Animals and other organisms
☒	☐ Clinical data
☒	☐ Dual use research of concern
☐	☒ Plants

Methods

n/a	Involved in the study
☒	☐ ChIP-seq
☒	☐ Flow cytometry
☒	☐ MRI-based neuroimaging

Antibodies

Antibodies used

HA and Flag monoclonal antibody (Abcam, Shanghai, China , AB 9110 and AB205606)

Validation

HA antibody:https://www.abcam.cn/products/primary-antibodies/ha-tag-antibody-chip-grade-ab9110.html
Flag antibody:https://www.abcam.cn/products/primary-antibodies/ddddk-tag-binds-to-flag-tag-sequence-antibody-epr20018-251-ab205606.html

Plants

Seed stocks

All seeds used and listed in Supplementary Table 1 in this study were maintained at the Cytogenetics Institute, Nanjing Agricultural University (CINAU).

Novel plant genotypes

In this study, we obtained the Pm55a and Pm55b transgenetic lines, and knock out SuPm55 in wheat plant. To produce transgenetic plants, the full-length Pm55a and Pm55b genomic sequences with native promoters were transformed separately into susceptible wheat cv. Fielder by Agrobacterium-mediated transformation. A total of 15 and 11 positive T0 transgenic plants with Pm55a and Pm55b, respectively, were obtained. To knock out SuPm55, the CRISPR/Cas9-mediated genome editing technology was employed. We used guide RNAs (gRNAs) targeting the conserved regions in CC domain of SuPm55, and obtained four mutant lines with 4-bp (Del5VS-1), 5-bp (Del5VS-2), 6-bp (Del5VS-3) and 27-bp (Del5VS-4) deletions in the target region as putative knockouts of the SuPm55 gene. In addition, 10 susceptible M1 mutants of TF5V-1 were identified by EMS, and three susceptible M1 mutants of NAU1908 were obtained. A novel line NAU2021 pyramiding Pm55a and Pm55b was produced by the hybridyzation of T5AL·5V#4S translocation line NAU185 (SuPm55/Pm55a) with the T5DL·5V#5S translocation line NAU1908 (Pm55b).

Authentication

The specific markers of Pm55a and Pm55b were used to identify the T0, T1 and T2 Fielder transgenetic progenies. We employed qRT-PCR analysis to verify the expression of Pm55a and Pm55b in T0 transgenic Fielder lines. To knock out SuPm55, we used the PCR to amplify the DNA sequence of SuPm55 in the T1 and T2 CRISPR/Cas9 edited progenies. The combination of GISH/FISH technologies were used to detect the F2 plants of NAU185/NAU1908 for the homozygous lines with Pm55a and Pm55b.
